# Protective Effects of Carnosic Acid on Lipopolysaccharide-Induced Acute Kidney Injury in Mice

**DOI:** 10.3390/molecules26247589

**Published:** 2021-12-14

**Authors:** Jung-Yeon Kim, Hyo-Lim Hong, Gyun Moo Kim, Jaechan Leem, Hyun Hee Kwon

**Affiliations:** 1 Department of Immunology, Daegu Catholic University School of Medicine, Daegu 42472, Korea; jy1118@cu.ac.kr; 2 Department of Internal Medicine, Daegu Catholic University School of Medicine, Daegu 42472, Korea; hlhong@cu.ac.kr; 3 Department of Emergency Medicine, Daegu Catholic University School of Medicine, Daegu 42472, Korea; emprof@cu.ac.kr

**Keywords:** sepsis, carnosic acid, acute kidney injury, lipopolysaccharide

## Abstract

Septic acute kidney injury (AKI) is an important medical problem worldwide, but current treatments are limited. During sepsis, lipopolysaccharide (LPS) activates various signaling pathways involved in multiorgan failure. Carnosic acid is a natural phenolic diterpene and has multiple bioactivities, such as anti-tumor, anti-inflammatory, and anti-oxidative effects. However, the effect of carnosic acid on septic AKI has not been explored. Therefore, this study aimed to determine whether carnosic acid has a therapeutic effect on LPS-induced kidney injury. Administration of carnosic acid after LPS injection ameliorated histological abnormalities and renal dysfunction. Cytokine production, immune cell infiltration, and nuclear factor-κB activation after LPS injection were also alleviated by carnosic acid. The compound suppressed oxidative stress with the modulation of pro-oxidant and antioxidant enzymes. Tubular cell apoptosis and caspase-3 activation were also inhibited by carnosic acid. These data suggest that carnosic acid ameliorates LPS-induced AKI via inhibition of inflammation, oxidative stress, and apoptosis and could serve as a useful treatment agent for septic AKI.

## 1. Introduction

Sepsis is a clinical syndrome of systemic inflammatory responses to infection and can lead to multiorgan failure [1]. Acute kidney injury (AKI) is the most serious and common complication of sepsis [2]. Importantly, septic AKI is the primary cause of death in hospitalized patients [2]. Current management of septic AKI consists of early administration of antibiotics, use of appropriate vasopressor agents, and fluid resuscitation [3]. However, the current treatment is reactive and nonspecific and its effectiveness is not sufficient [3]. Therefore, the development of new, effective strategies or medications for septic AKI is of great clinical importance. Despite many research efforts, the pathogenesis of septic AKI is not fully understood. However, accumulating evidence suggests that multiple mechanisms, including inflammation, oxidative stress, and apoptosis, are interconnected in the underlying pathophysiology of septic AKI [3,4,5]. Lipopolysaccharide (LPS), also referred to as endotoxin, is an important bacterial component [3]. During sepsis, LPS is released into the systemic circulation and binds to Toll-like receptors (TLRs), activating several signaling pathways involved in multiorgan failure [3,4,5].

Natural products have received much attention as a promising source of bioactive compounds that are potentially useful for drug discovery [6]. Many natural product-derived compounds, such as resveratrol, curcumin, and luteolin, have anti-inflammatory, anti-oxidative, and anti-apoptotic properties [7,8]. These compounds have been shown to exert beneficial effects on septic AKI [9,10,11]. Carnosic acid is a phenolic diterpene found in rosemary [12]. This compound possesses anti-tumor, anti-inflammatory, and anti-oxidative activities [13]. Previous studies have shown that carnosic acid exerts protective effects against cisplatin- or cadmium-induced AKI [14,15]. Diabetes-related kidney injury and unilateral ureteral-obstruction-induced kidney injury have also been shown to be attenuated by carnosic acid [16,17]. However, the effect of carnosic acid on septic AKI remains undetermined. Animal models of LPS-induced AKI have been widely used to discover new therapeutic agents for septic AKI or to investigate the underlying mechanisms [3]. Therefore, this study aimed to determine whether carnosic acid has a therapeutic effect on endotoxin-induced kidney injury. In addition, the effect of the compound on inflammation, oxidative stress, and apoptosis was examined.

## 2. Results

### 2.1. Carnosic Acid Ameliorated Endotoxin-Induced AKI

**Figure 1:**
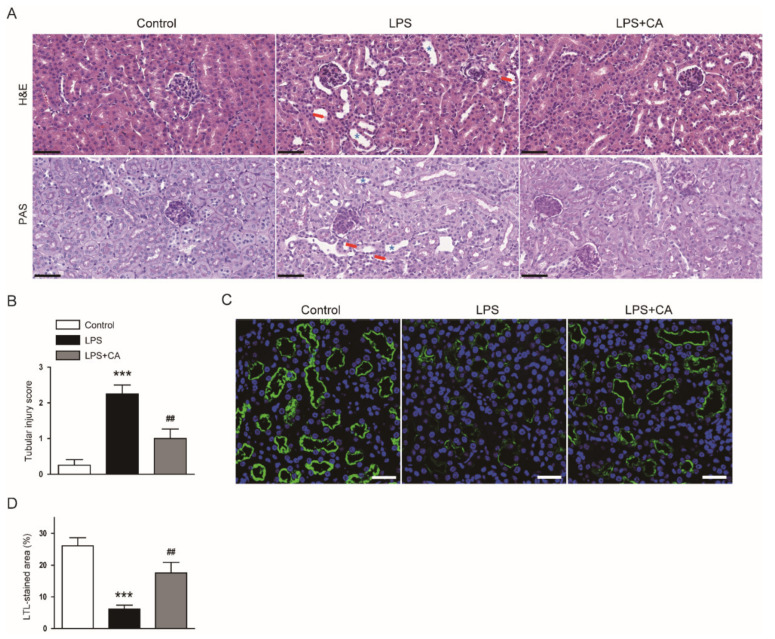
Effect of carnosic acid on histopathological features. (**A**) H&E and PAS staining. Scale bar: 50 μm. Red arrows indicate tubular cell detachment. Blue asterisks indicate dilated tubules. (**B**) Tubular injury score. (**C**) IF staining for LTL (green) on kidney sections. To stain nuclei, DAPI (blue) was used. Scale bar: 40 μm. (**D**) Percentage of LTL-stained cells. *n* = 8 per group of mice. *** *p* < 0.001 versus control. ^#^^#^ *p* < 0.01 versus LPS.

To determine whether carnosic acid exerts a therapeutic action on endotoxin-induced kidney injury, we first examined the effect of carnosic acid on histological abnormalities in LPS-injected mice. On histological examination, pathological features, such as tubular cell detachment and tubular dilatation, were observed in endotoxin-injected mice (Figure 1A,B). However, these pathological changes were ameliorated by carnosic acid (*p* < 0.01; Figure 1A,B). Lotus tetragonolobus lectin (LTL) is widely used for labeling the brush border of the tubule [18]. We next performed immunofluorescence (IF) staining with LTL on kidney sections to explore the effect of carnosic acid on brush border membranes. Carnosic acid attenuated brush border loss in endotoxin-injected mice, as evidenced by the increased percentage of the LTL-stained area (*p* < 0.01; Figure 1C,D).

**Figure 2:**
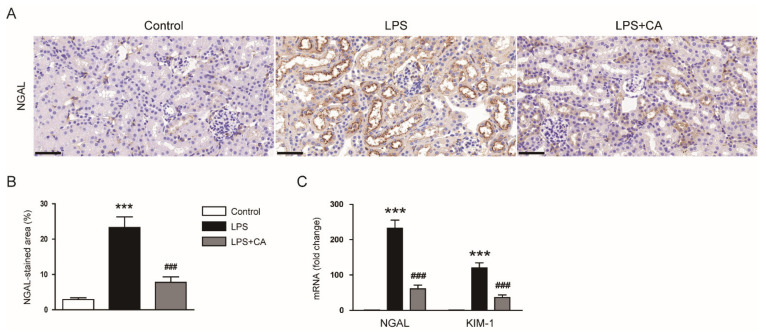
Effect of carnosic acid on tubular injury markers. (**A**) IHC staining for NGAL on kidney sections. Scale bar: 50 μm. (**B**) Percentage of NGAL-stained area. (**C**) The mRNA expression of NGAL and KIM-1 in kidney tissues. *n* = 8 per group. *** *p* < 0.001 versus control. ^#^^#^^#^ *p* < 0.001 versus LPS.

To further examine the effect of carnosic acid on tubular injury, immunohistochemistry (IHC) staining for neutrophil-gelatinase-associated lipocalin (NGAL), a tubular injury marker [19], was performed. The percentage of NGAL-stained area was higher in the LPS group than in the control group (Figure 2A,B). LPS injection also increased mRNA levels of NGAL and kidney injury molecule-1 (KIM-1) (Figure 2C). However, carnosic acid reduced the expression of the markers (*p* < 0.001; Figure 2A–C).

**Figure 3:**
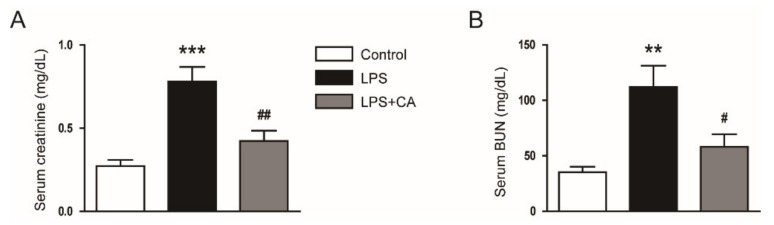
Effect of carnosic acid on serum creatinine and BUN levels. (**A**) Serum creatinine levels. (**B**) Serum BUN levels. *n* = 8 per group. ** *p* < 0.01 and *** *p* < 0.001 versus control. ^#^ *p* < 0.05 and ^##^ *p* < 0.01 versus LPS.

Serum creatinine and blood urea nitrogen (BUN) levels are indicators of renal function [20]. The levels of these indicators were higher in the LPS group than in the control group (Figure 3A,B). However, carnosic acid reduced the elevation of serum creatinine (*p* < 0.01) and BUN (*p* < 0.05) levels (Figure 3A,B). Altogether, these findings indicate that carnosic acid ameliorated LPS-induced tubular injury and renal dysfunction.

### 2.2. Carnosic Acid Attenuated LPS-Induced Inflammatory Responses

**Figure 4:**
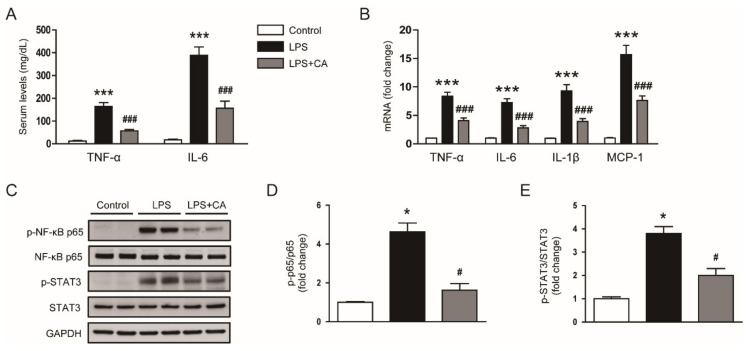
Effect of carnosic acid on cytokine production. (**A**) Serum TNF-α and IL-6 levels. (**B**) The mRNA expression of TNF-α, IL-6, IL-1β, and MCP-1 in kidney tissues. (**C**) Western blotting of p-NF-κB p65 and p-STAT3 in kidney tissues. (**D**) Quantification of Western blot data for p-NF-κB p65. (**E**) Quantification of Western blot data for p-STAT3. *n* = 8 per group. * *p* < 0.05 and *** *p* < 0.001 versus control. ^#^ *p* < 0.05 and ^#^^#^^#^ *p* < 0.001 versus LPS.

Excessive production of cytokines and massive infiltration of immune cells are common pathological features of AKI [21]. Thus, we assessed the effect of carnosic acid on cytokine production and immune cell infiltration in endotoxin-injected mice. Serum tumor necrosis factor-α (TNF-α) and interleukin-6 (IL-6) levels were higher in the LPS group than in the control group (Figure 4A). Hepatic mRNA levels of TNF-α, IL-6, IL-1β, and monocyte chemoattractant protein-1 (MCP-1) were also increased after LPS treatment (Figure 4B). However, carnosic acid inhibited the LPS-induced cytokine production (*p* < 0.001; Figure 4A,B). Furthermore, phosphorylated forms of nuclear factor-κB (NF-κB) p65 and signal transducer and activator of transcription 3 (STAT3) were increased after LPS injection, which was inhibited by carnosic acid (*p* < 0.05; Figure 4C–E).

**Figure 5:**
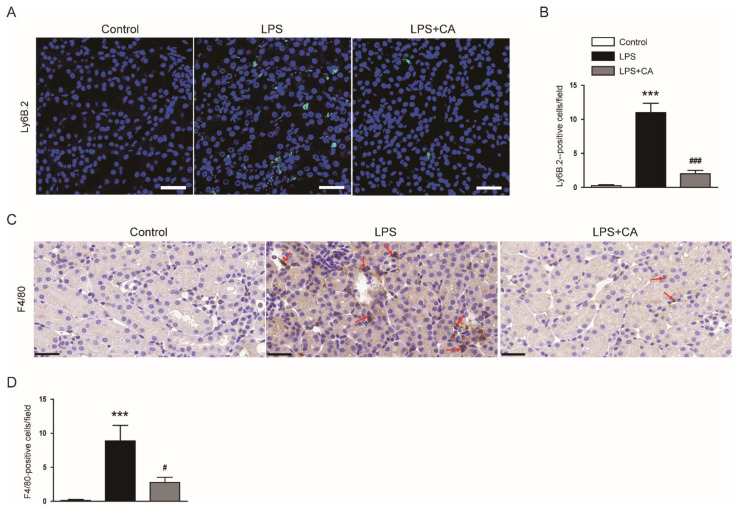
Effect of carnosic acid on neutrophil and macrophage infiltration. (**A**) IF staining for Ly6B.2 (green) on kidney sections. To stain nuclei, DAPI (blue) was used. Scale bar: 40 μm. (**B**) Number of Ly6B.2-positive cells. (**C**) IHC staining for F4/80 on kidney sections. Red arrows indicate positively stained cells. Scale bar: 30 μm. (**D**) Number of F4/80-stained cells per field. *n* = 8 per group of mice. ^#^ *p* < 0.05 and *** *p* < 0.001 versus control. ^#^^#^^#^ *p* < 0.001 versus LPS.

Neutrophil infiltration was evaluated by detecting cells stained with Ly6B.2, a neutrophil marker [22]. The number of Ly6B.2-positive cells was higher in the LPS group than in the control group (Figure 5A,B). However, the LPS-induced neutrophil infiltration was inhibited by carnosic acid (*p* < 0.001; Figure 5A,B).

We also performed IHC staining for F4/80, a macrophage marker [23], on kidney sections to examine macrophage infiltration into the kidney. The number of F4/80-positive cells was increased after LPS injection, which was reduced by carnosic acid (*p* < 0.05; Figure 5C,D). Altogether, these data indicate that carnosic acid attenuated inflammatory responses in LPS-injected mice.

### 2.3. Carnosic Acid Alleviated Endotoxin-Induced Oxidative Stress

**Figure 6:**
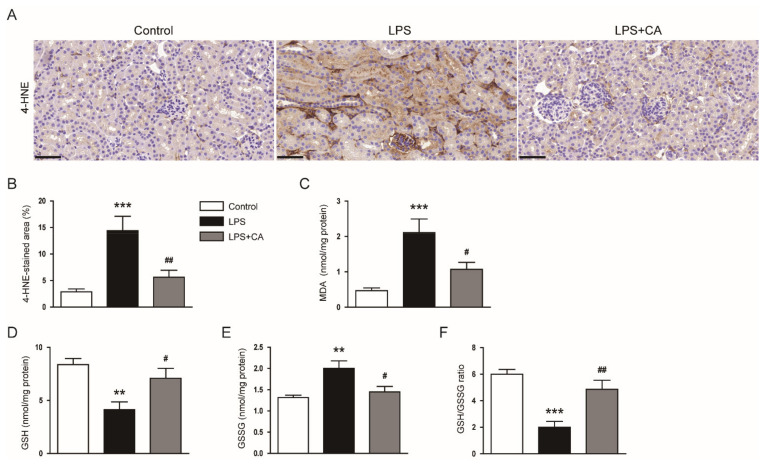
Effect of carnosic acid on oxidative stress. (**A**) IHC staining for 4-HNE. Scale bar = 50 μm. (**B**) Percentage of 4-HNE-stained area. (**C**) Renal MDA levels. *n* = 8 per group of mice. (**D**) Renal GSH levels. (**E**) Renal GSSG levels. (**F**) GSH/GSSG ratios. ** *p* < 0.01 and *** *p* < 0.001 versus control. ^#^ *p* < 0.05 and ^#^^#^ *p* < 0.01 versus LPS.

Oxidative stress plays a key role in the pathogenesis of septic AKI [4]. Because carnosic acid has been shown to have potent anti-oxidative properties [24,25], the levels of lipid peroxidation by-products [26], 4-hydroxynonenal (4-HNE) and malondialdehyde (MDA), were examined. IHC staining showed that the 4-HNE-stained area was larger in the LPS group than in the control group (Figure 6A,B). Hepatic MDA levels were also increased after LPS treatment (Figure 6C). However, carnosic acid reduced the amounts of 4-HNE (*p* < 0.01) and MDA (*p* < 0.05) (Figure 6A–C).

In addition to lipid peroxidation, the ratio of reduced glutathione (GSH) to oxidized GSH (GSSG) was analyzed to assess oxidative stress. Renal GSH levels were lower in the LPS group than in the control group, but GSSG levels were increased after LPS treatment (Figure 6D,E). However, carnosic acid attenuated all these changes (*p* < 0.05; Figure 6D,E). AS a result, a reduction in the GSG/GSSG ratio was also reversed by carnosic acid (*p* < 0.01; Figure 6F).

**Figure 7:**
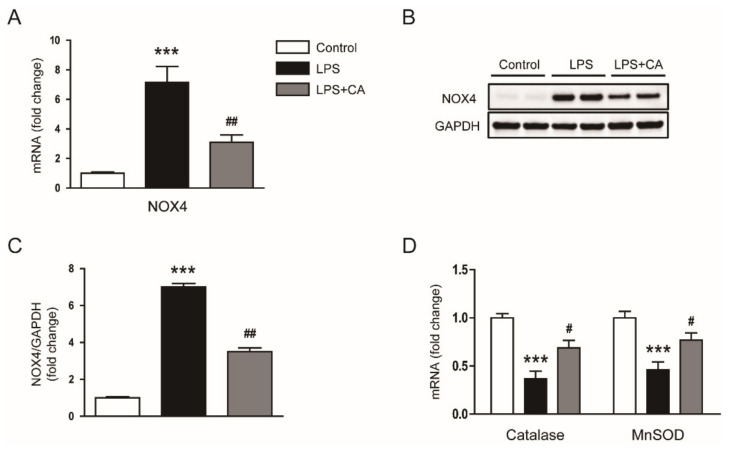
Effect of carnosic acid on the expression of NOX4, catalase, and MnSOD. (**A**) The mRNA expression of NOX4 in kidney tissues. (**B**) Western blotting of NOX4 in kidney tissues. (**C**) Quantification of Western blot data for NOX4. (**D**) The mRNA expression of catalase and MnSOD in kidney tissues. *** *p* < 0.001 versus control. ^#^ *p* < 0.05 and ^#^^#^ *p* < 0.01 versus LPS.

Pro-oxidant and antioxidant systems play major roles in the modulation of oxidative stress [27,28]. Recently, nicotinamide adenine dinucleotide phosphate oxidase 4 (NOX4) was identified as a major source of reactive oxygen species (ROS) production in endotoxin-induced AKI [29,30]. We confirmed that NOX4 expression was higher in the LPS group than in the control group (Figure 7A–C). However, the increased expression of NOX4 mRNA and protein was inhibited by carnosic acid (*p* < 0.01; Figure 7A–C). Moreover, carnosic acid reversed the decreased expression of catalase and manganese superoxide dismutase (MnSOD) (*p* < 0.05; Figure 7D).

### 2.4. Carnosic Acid Inhibited Endotoxin-Induced Tubular Cell Apoptosis

**Figure 8:**
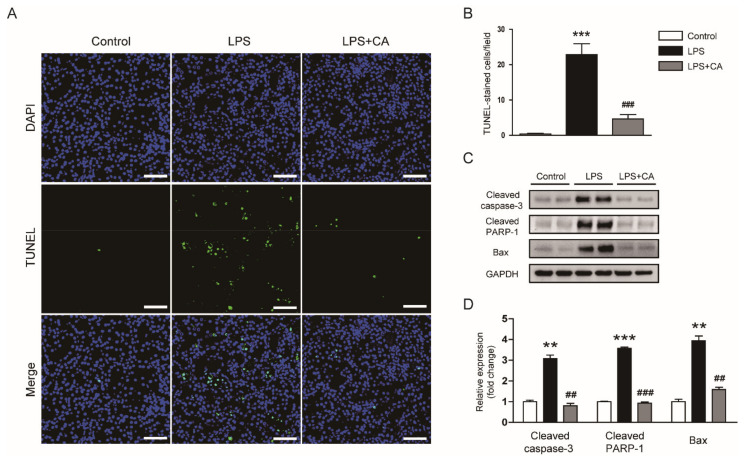
Effect of carnosic acid on apoptosis and caspase-3 activation. (**A**) TUNEL staining. Scale bar: 100 μm. (**B**) Number of TUNEL-stained cells per field. (**C**) Western blotting of cleaved caspase-3, cleaved PARP-1, and Bax. (**D**) Quantification of Western blot data for cleaved caspase-3, cleaved PARP-1, and Bax. ** *p* < 0.01 and *** *p* < 0.001 versus control. ^#^^#^ *p* < 0.01 and ^#^^##^ *p* < 0.001 versus LPS.

Tubular cell apoptosis is also involved in the pathophysiology of septic AKI [5]. Therefore, we carried out TdT-mediated dUTP nick end labeling (TUNEL) staining to detect apoptotic cells. The number of apoptotic cells was higher in the LPS group than in the control group (Figure 8A,B). However, carnosic acid significantly inhibited tubular cell apoptosis in LPS-injected mice (*p* < 0.001; Figure 8A,B). Protein levels of cleaved caspase-3, cleaved poly(ADP-ribose) polymerase-1 (PARP-1), and Bax were also reduced by carnosic acid (*p* < 0.01 for cleaved caspase-3 and Bax; *p* < 0.001 for cleaved PARP-1; Figure 8C,D).

## 3. Discussion

The aim of the current study was to determine the effect of carnosic acid on LPS-induced kidney injury. Our data demonstrated that administration of carnosic acid ameliorates LPS-induced tubular injury and renal dysfunction. Cytokine production and immune cell infiltration after LPS treatment were reduced by carnosic acid. The compound alleviated LPS-induced oxidative stress via regulation of pro-oxidant and antioxidant enzymes. Tubular cell apoptosis and caspase-3 activation were also inhibited by carnosic acid.

Carnosic acid is a natural phenolic diterpene with anti-oxidative and anti-inflammatory properties [12,13]. Previous animal studies have reported that carnosic acid is beneficial for various inflammatory diseases, such as myocardial ischemia-reperfusion injury [31], hepatic ischemia-reperfusion injury [32], acetaminophen-induced hepatotoxicity [33], non-alcoholic fatty liver disease [34], rheumatoid arthritis [35], Parkinson’s disease [36], and inflammatory bowel disease [37]. In this study, we showed that carnosic acid ameliorates tubular injury in endotoxin-injected mice, as evidenced by improvement in tubular injury score, reduction in the loss of LTL-stained brush border, and downregulation of NGAL and KIM-1. In addition, carnosic acid reduced serum creatinine and BUN levels in LPS-injected mice. Taken together, these results indicate that carnosic acid inhibits LPS-induced structural and functional damage to the kidney.

During sepsis, excessive activation of the innate immune system can cause systemic inflammatory responses, resulting in aggravation of tissue injury [38,39]. LPS is one of the most well-known PAMPs and interacts with TLRs to activate multiple signaling cascades involved in multiorgan failure [3]. In this study, LPS treatment increased serum and renal levels of cytokines and induced infiltration of Ly6B.2-postive neutrophils and F4/80-positive macrophages into the kidney. However, these inflammatory responses were significantly inhibited by carnosic acid. In addition, the inhibitory effect of the compound on LPS-induced inflammation was associated with attenuation of NF-κB activation. It is well known that the TLR4-NF-κB pathway plays a critical role in LPS-induced inflammation [3]. Therefore, our findings suggest that carnosic acid ameliorates LPS-induced inflammation, probably by inhibiting the TLR4-NF-κB pathway. Consistent with these results, Li et al. showed that carnosic acid protects against endotoxin-induced lung injury in mice via the inhibition of the TLR4-NF-κB pathway [40]. Xiang et al. showed that carnosic acid attenuates endotoxin-induced liver injury in rats [41]. In vitro studies have shown that carnosic acid inhibits NF-κB pathway and cytokine production in LPS-stimulated human keratinocyte cells [42] and mouse adipocytes [43]. Endotoxin-induced activation of mouse microglial cells was also alleviated by carnosic acid [44]. In addition, STAT3 signaling is known to have a cross-talk with NF-κB signaling and plays an important role in the inflammatory response [45]. In this study, we found that carnosic acid also significantly inhibits STAT3 activation. Consistently, a previous study reported that carnosic acid inhibits STAT3 signaling to suppress chemokine production in IL-27-treated human oral epithelial cells [46]. The compound also exerts anti-inflammatory effects in human periodontal ligament cells through inhibiting NF-κB and STAT3 cascades [47].

Oxidative stress is critically involved in the pathophysiology of septic AKI [4]. Our data showed marked induction of oxidative stress by endotoxin, as evidenced by increased lipid peroxidation and a decreased GSG/GSSG ratio. However, carnosic acid significantly attenuated LPS-induced oxidative stress. Indeed, carnosic acid is known to have potent anti-oxidative activity, which is consistent with our results [24,25]. The compound exerts anti-oxidative effects to ameliorate inflammatory diseases, such as hepatic ischemia-reperfusion injury [32], acetaminophen-induced hepatotoxicity [33], Parkinson’s disease [36], and inflammatory bowel disease [37]. To further investigate the mechanisms, we analyzed the expression of pro-oxidant and antioxidant enzymes. LPS treatment increased NOX4 expression and decreased catalase and MnSOD expression but was significantly reversed by carnosic acid. NOX4 plays a major role in ROS production in LPS-induced AKI [29,30]. Although the detailed molecular mechanism is not yet fully understood, several studies have shown the inhibitory action of carnosic acid on NOX4 expression [17,48,49]. In addition, upregulation of antioxidant enzymes by carnosic acid was observed in cisplatin-induced nephrotoxicity [14]. Therefore, these findings suggest that carnosic acid attenuates LPS-induced oxidative stress via regulation of pro-oxidant and antioxidant enzymes.

Besides inflammation and oxidative stress, tubular cell apoptosis also plays an important role in septic AKI [5]. Indeed, apoptotic tubular epithelial cells are frequently observed in mice and patients with septic AKI [50,51,52,53]. In this study, LPS injection increased tubular cell apoptosis and caspase-3 activation. Protein levels of Bax, a proapoptotic molecule, were also increased. However, these pathological processes were significantly inhibited by carnosic acid. Many studies have shown that the compound induces apoptosis in cancer cells [54] However, some studies have revealed the anti-apoptotic effect of carnosic acid on normal cells in inflammatory conditions [55,56,57,58,59]. In animal models of several inflammatory diseases, carnosic acid inhibited apoptosis to ameliorate inflammation and tissue injury [48,59,60]. Thus, the cytoprotective effect of carnosic acid may contribute to its therapeutic action on LPS-induced AKI.

## 4. Materials and Methods

### 4.1. Animals and Treatment

Male C57BL/6 mice (7 weeks of age) were acquired from HyoSung Science Inc. (Daegu, Korea). The mice were kept under controlled conditions in a light/dark cycle of 12 h/12 h, a temperature of 20–24 °C, and humidity of 60–70%. After 1 week of adaptation, the mice were randomly divided into three groups, with 8 mice in each group: the control group, the LPS group, and the LPS+CA group. The mice in the LPS group were given a single intraperitoneal injection of LPS (Sigma-Aldrich, St. Louis, MO, USA) at a dose of 10 mg/kg. The LPS+CA group was intraperitoneally injected with carnosic acid (dissolved in dimethyl sulfoxide (DMSO); Santa Cruz Biotechnology, Santa Cruz, CA, USA) at a dose of 40 mg/kg, 1 h after LPS injection. The control group received an equal volume of DMSO. All mice were anesthetized and sacrificed 24 h after LPS injection. Blood and kidney tissues were immediately collected. The doses of carnosic acid and LPS were selected based on previous studies [40,61].

### 4.2. Assessment of Renal Function and Oxidative Stress

Serum creatinine and BUN levels were analyzed using an automatic analyzer (Hitachi, Osaka, Japan). Renal MDA levels were measured using an MDA assay kit (Sigma-Aldrich, St. Louis, MO, USA) following the manufacturer’s protocol. Renal GSH and GSSG levels were assessed using a GSH assay kit (Enzo Life Sciences, Farmingdale, NY, USA) following the manufacturer’s instructions.

### 4.3. Measurement of Serum Cytokines

Serum TNF-α and IL-6 levels were measured using ELISA kits (R&D Systems, Minneapolis, MN, USA) following the manufacturer’s protocol.

### 4.4. Histological Analysis and IHC Staining

Kidney tissues were fixed, dehydrated, cleared, and embedded in paraffin. The paraffin blocks were sectioned, stained with hematoxylin and eosin (H&E) or periodic acid-Schiff (PAS), and viewed under a slide scanner (3DHISTECH, Budapest, Hungary). The percentage of damaged area was evaluated to assess the severity of tubular injury: 0, 0%; 1, ≤10%; 2, 11–25%; 3, 26–45%; 4, 46–75%; and 5, 76–100% [62,63]. Tubular injury was examined in 5 arbitrarily chosen fields per sample. For IHC, sections were immunostained with primary antibodies against NGAL (Santa Cruz Biotechnology, Santa Cruz, CA, USA), F4/80 (Santa Cruz Biotechnology), and 4-HNE (Abcam, Cambridge, MA, USA). Then, the slides were probed with secondary antibodies. The percentage of areas stained with NGAL or 4-HNE was analyzed using the i-Solution DT software (IMT i-Solution Inc., Coquitlam, BC, Canada). Five arbitrarily chosen fields per sample were examined. The F4/80-positive cells were examined in 10 arbitrarily chosen fields per sample.

### 4.5. IF Staining

Sections were deparaffinized, dehydrated, and blocked in a blocking buffer. The sections were probed with an anti-Ly6B.2 antibody (Abcam). After washing, the sections were probed with an Alexa-Fluor-488-conjugated secondary antibody (Invitrogen, Carlsbad, CA, USA). Nuclear staining was performed with DAPI. The brush border of the tubules was detected using fluorescein-5-isothiocyanate (FITC)-conjugated LTL (Vector Laboratories, Burlingame, CA, USA). Images were taken with a confocal microscope (Nikon, Tokyo, Japan). Positive cells were examined in 10 arbitrarily selected fields per sample.

### 4.6. Western Blot Analysis

The total protein in the kidney was extracted using a lysis buffer (Sigma-Aldrich, St. Louis, MO, USA). Protein samples were loaded onto polyacrylamide gels and transferred to nitrocellulose membranes. The membranes were probed with primary antibodies against p-NF-κB p65 (Cell Signaling, Danvers, MA, USA), NF-κB p65 (Cell Signaling), p-STAT3 (Cell Signaling), STAT3 (Cell Signaling), NOX4 (Novus Biologicals, Littleton, CO, USA), cleaved caspase-3 (Cell Signaling), cleaved PARP-1 (Cell Signaling), Bax (Santa Cruz Biotechnology), and glyceraldehyde-3-phosphate dehydrogenase (GAPDH; Cell Signaling). After washing, the membranes were incubated with secondary antibodies. Quantification of Western blot data was conducted using ImageJ software (National Institutes of Health, Bethesda, MD, USA).

### 4.7. Real-Time Reverse Transcription Polymerase Chain Reaction (RT-PCR)

The TRIzol reagent (Sigma-Aldrich, St. Louis, MO, USA) was used for the extraction of total RNA from kidney tissues. Total RNA was reversely transcribed into cDNA using the PrimeScript RT Reagent Kit (TaKaRa, Tokyo, Japan). Real-time RT-PCR was conducted using Power SYBR Green PCR Master Mix (Thermo Fisher Scientific) in Thermal Cycler Dice Real Time System III (TaKaRa). Primers are listed in Table 1. GAPDH was used as an internal reference. Data were analyzed using the delta-delta CT method.

### 4.8. TUNEL Assay

Apoptotic cells were detected using a TUNEL assay kit (Roche Diagnostics, Indianapolis, IN, USA) following the manufacturer′s protocol. Positive cells were examined in 10 arbitrarily selected fields per sample.

### 4.9. Statistical Analysis

Data were expressed as the mean ± SEM. Differences among the groups were analyzed with one-way ANOVA and Bonferroni’s post hoc tests. A *p*-value less than 0.05 was considered statistically significant.

## 5. Conclusions

In conclusion, we demonstrated that carnosic acid has a therapeutic effect on endotoxin-induced AKI, as evidenced by improvement in histological abnormalities and reduction in serum creatinine and BUN levels. NF-κB-mediated inflammatory responses and caspase-3-dependent apoptosis in LPS-induced AKI was attenuated by carnosic acid. The compound also inhibited LPS-induced oxidative stress via regulation of pro-oxidant and antioxidant enzymes. These results suggest that carnosic acid could serve as a potential treatment option for septic AKI.

## Figures and Tables

**Table 1:** List of primers.

Gene	Primer Sequence (5′→3′)	Accession No.
NGAL	Forward: GACCTAGTAGCTGCTGAAACC Reverse: GAGGATGGAAGTGACGTTGTAG	NM_130741
KIM-1	Forward: TCCACACATGTACCAACATCAA Reverse: GTCACAGTGCCATTCCAGTC	NM_001161356
TNF-α	Forward: GACGTGGAACTGGCAGAAGAG Reverse: CCGCCTGGAGTTCTGGAA	NM_013693
IL-6	Forward: CCAGAGATACAAAGAAATGATGG Reverse: ACTCCAGAAGACCAGAGGAAAT	NM_031168
IL-1β	Forward: GCAACTGTTCCTGAACTCAACT Reverse: ATCTTTTGGGGTCCGTCAACT	NM_008361
MCP-1	Forward: TAAAAACCTGGATCGGAACCAA Reverse: GCATTAGCTTCAGATTTACGGGT	NM_011333
NOX4	Forward: GAACCCAAGTTCCAAGCTCATT Reverse: GGCACAAAGGTCCAGAAATCC	NM_015760
Catalase	Forward: CAAGTACAACGCTGAGAAGCCTAAG Reverse: CCCTTCGCAGCCATGTG	NM_009804
MnSOD	Forward: AACTCAGGTCGCTCTTCAGC Reverse: CTCCAGCAACTCTCCTTTGG	NM_013671
GAPDH	Forward: ACTCCACTCACGGCAAATTC Reverse: TCTCCATGGTGGTGAAGACA	NM_001289726

## Data Availability

The data supporting the findings of this study are available within the article.

## Sample Availability

Samples of the compounds are not available from the authors.
